# Supplementary material for: Genomic and transcriptomic changes complement each other in the pathogenesis of sporadic Burkitt lymphoma
Source: Nat Commun. 2019 Mar 29;10:1459. doi: 10.1038/s41467-019-08578-3 (PMC6440956; doi:10.1038/s41467-019-08578-3)
Supplement: Supplementary file 1 — Supplementary Information [file 41467_2019_8578_MOESM1_ESM.pdf]

Supplementary Information to Genomic and transcriptomic changes complement each other in the pathogenesis of sporadic Burkitt Lymphoma

López, Kleinheinz, Aukema, Rohde, Bernhart, Hübschmann, Wagener, Toprak et al.. 2018

Table of contents:

1. Supplementary Note 1:

   Full list of members of the Deutsche Krebshilfe Network Project

   "Molecular Mechanisms in Malignant Lymphoma" (MMML) (alphabetical order) :3

2. Supplementary Figures :4-16

3. Supplementary Table :17

4. References :18

Supplementary Note 1.

Full list of members of the MMML

Pathology group and analytes preparation: Thomas F.E. Barth[1], Heinz-Wolfram Bernd[2], Sergio B. Cogliatti[3], Alfred C. Feller[2], Martin L. Hansmann[4], Michael Hummel[5], Wolfram Klapper[6], Dido Lenze[5], Peter Möller[1], Hans-Konrad Müller-Hermelink[7], German Ott[7], Andreas Rosenwald[7], Harald Stein[5], Monika Szczepanowski[6], Hans-Heinrich Wacker[6].
Genetics group: Thomas F.E. Barth[1], Petra Behrmann[8], Peter Daniel[10], Judith Dierlammm8, Eugenia Haralambieva[7], Lana Harder[11], Paul-Martin Holterhus[12], Ralf Küppers[13], Dieter Kube[13], Peter Lichter[14], Jose I. Martín-Subero[11], Peter Möller[1], Eva M. Murga-Peñas[9], German Ott[7], Christiane Pott[16], Armin Pscherer[15], Andreas Rosenwald[7], Carsten Schwaenen[17], Reiner Siebert[18], Heiko Trautmann[16], Martina Vockerodt[19], Swen Wessendorf[16].
Bioinformatics group: Stefan Bentink[20], Hilmar Berger[21], Dirk Hasenclever[21], Markus Kreuz[21], Markus Loeffler[21], Maciej Rosolowski[21], Rainer Spang[20].
Project coordination: Benjamin Stürzenhofecker[14], Lorenz Trümper[14], Maren Wehner[14].
Steering committee: Markus Loeffler[21], Reiner Siebert[18], Harald Stein[5], Lorenz Trümper[14].

[1]Institute of Pathology, University Hospital of Ulm, Ulm, Germany;
[2]Institute of Pathology, University Hospital Schleswig-Holstein Campus Lübeck, Lübeck, Germany;
[3]Institute of Pathology, Kantonsspital St. Gallen, St.Gallen, Switzerland;
[4]Institute of Pathology, University Hospital of Frankfurt, Frankfurt, Germany;
[5]Institute of Pathology, Campus Benjamin Franklin, Charité–Universitätsmedizin Berlin, Berlin, Germany;
[6]Institute of Hematopathology, University Hospital Schleswig-Holstein Campus Kiel/ Christian-Albrechts University Kiel, Kiel, Germany;
[7]Institute of Pathology, University of Würzburg, Würzburg, Germany;
[8]Cytogenetic and Molecular Diagnostics, Internal Medicine III, University Hospital of Ulm, Ulm, Germany;
[9]University Medical Center Hamburg-Eppendorf, Hamburg, Germany;
[10]Department of Hematology, Oncology and Tumor Immunology, University Medical Center Charité, Berlin, Germany;
[11]Institute of Human Genetics, University Hospital Schleswig-Holstein Campus Kiel/Christian-Albrechts University Kiel, Kiel, Germany;
[12]Division of Pediatric Endocrinology and Diabetes, Department of Pediatrics, University Hospital Schleswig- Holstein Campus Kiel / Christian-Albrechts University Kiel, Kiel, Germany;
[13]Institute for Cell Biology (Tumor Research), University of Duisburg-Essen, Essen, Germany;
[14]Department of Hematology and Oncology, Georg-August University of Göttingen, Göttingen, Germany;
[15]German Cancer Research Center (DKFZ), Heidelberg, Germany;
[16]Second Medical Department, University Hospital Schleswig-Holstein Campus Kiel/ Christian-Albrechts University Kiel, Kiel, Germany;
[17]Cytogenetic and Molecular Diagnostics, Internal Medicine III, University Hospital of Ulm, Ulm, Germany;
[18]Institute of Human Genetics, University of Ulm and University Hospital of Ulm, Ulm, Germany
[19]Department of Pediatrics I, Georg-August University of Göttingen, Göttingen, Germany;
[20]Institute of Functional Genomics, University of Regensburg, Regensburg, Germany;
[21]Institute for Medical Informatics, Statistics and Epidemiology, University of Leipzig, Leipzig, Germany.

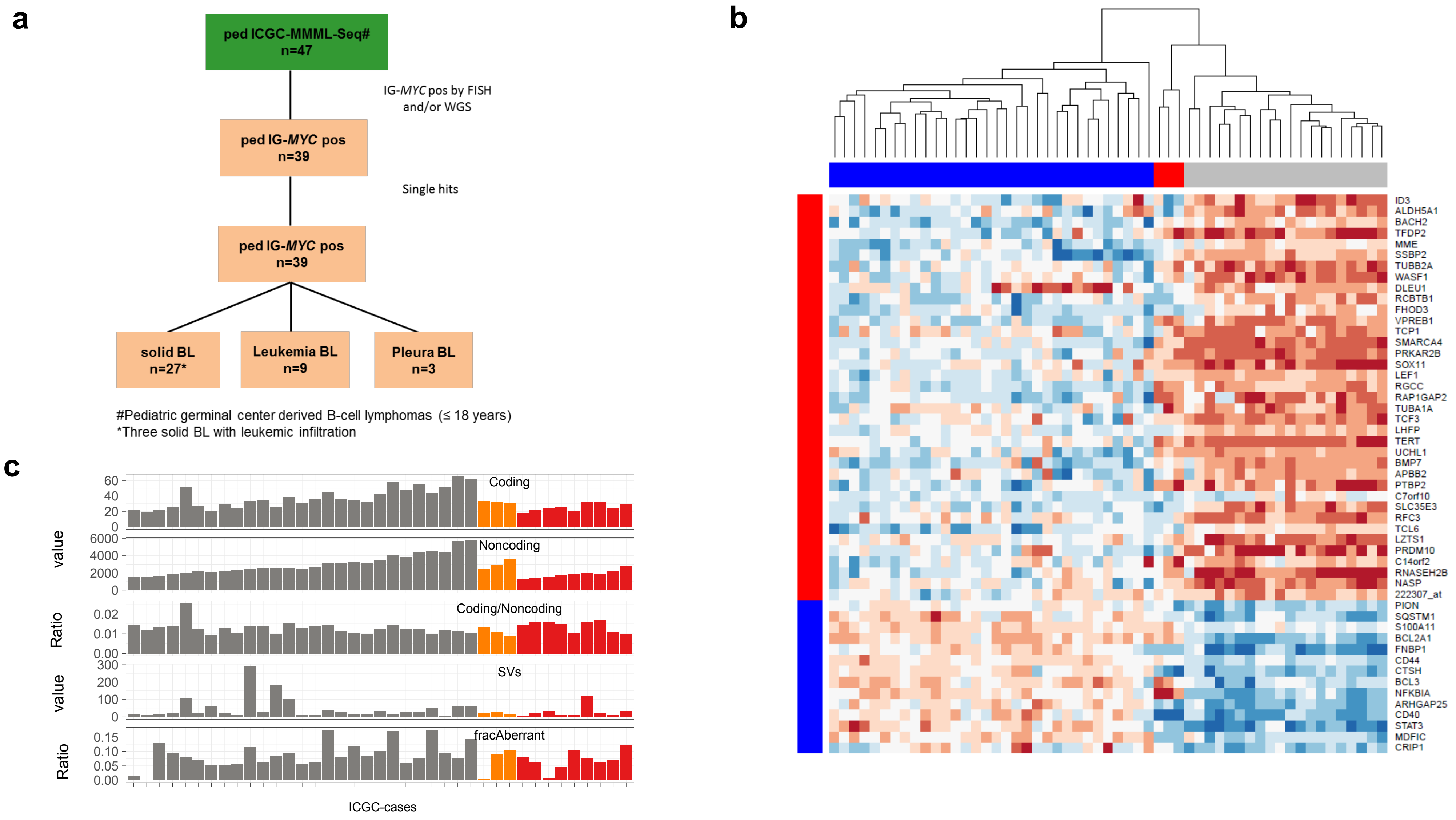

Supplementary Figure 1. Characteristics of the cohort and number of events detected by WGS.

a Inclusion criteria of the BL cases of the ICGC MMML-Seq cohort analyzed.

b The heatmap comprises all n=51 genes of the mBL vs. non-mBL expression classifier. Patients of the ICGC MMML-Seq cohort with available transcriptome data were clustered hierarchically and labelled: prototypic non-BL cases (blue), leukBL cases (red), and solBL cases (gray). Genes upregulated in BL are marked in red, genes downregulated in BL are marked in blue.

c Total number of coding and non-coding events (SNVs, indels, SVs) and fraction of aberrations in BL cohort (grey: solBL, orange: pleuraBL, and red: leukBL).

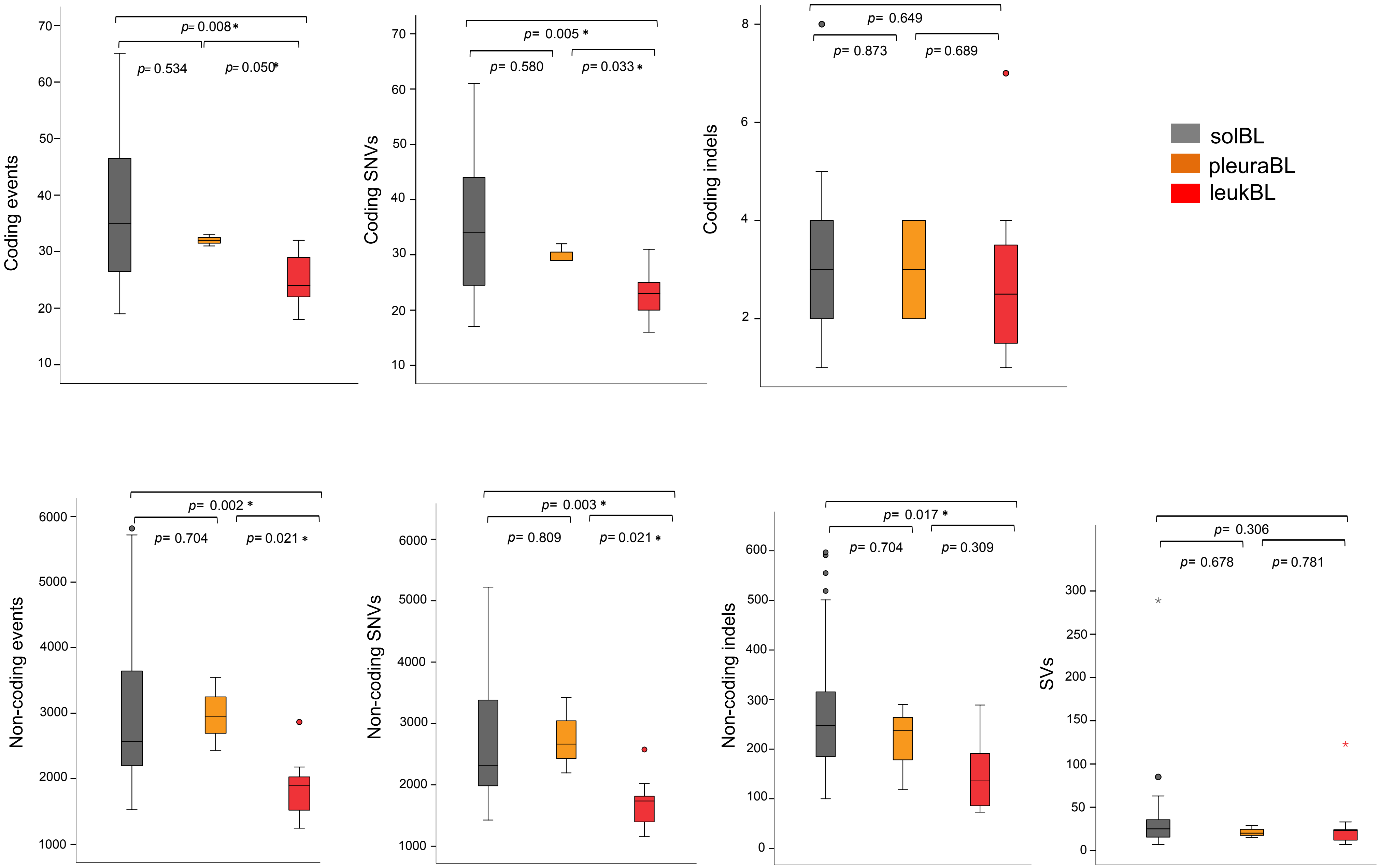

Supplementary Figure 2. Box-plot of genomic events detected by WGS in BL cohort.

The upper panel shows the total number of coding events, coding SNVs, and coding indels (upper part), distributed over the solBL (grey), pleuraBL (orange), leukBL (red). The lower panel shows the total number of non-coding events, non-coding SNVs, non-coding indels, as well as SVs across the cohort (labeled as described above). Statistical analyses were performed using Kruskal-Wallis test for all the variables described. Significance considered as p-value ≤0.05 is indicated by an asterisk (*).

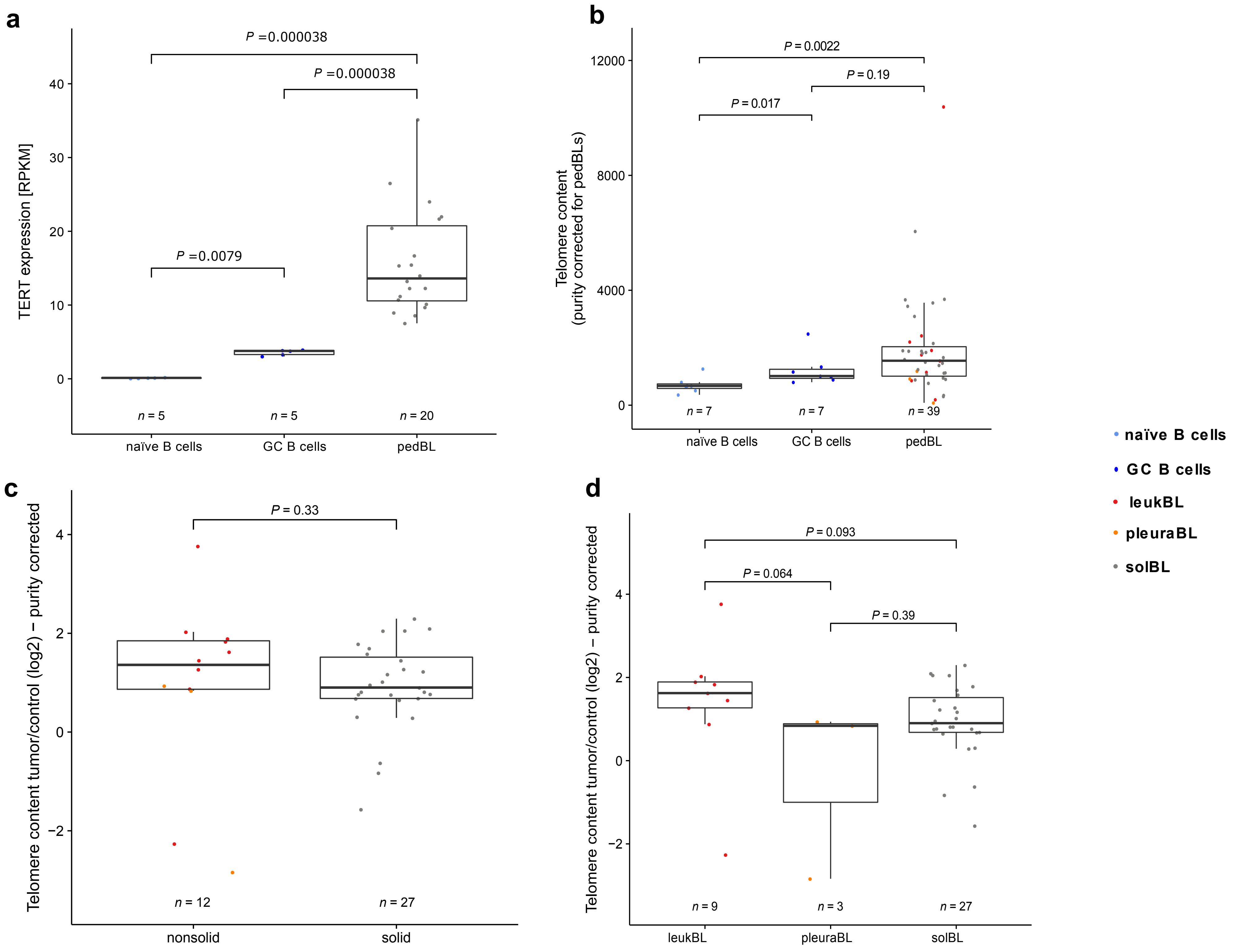
a
P =0.000038
P =0.000038
P =0.0079
TERT expression [RPKM]
n = 5
n = 5
n = 20
naïve B cells
GC B cells
pedBL
b
P = 0.0022
P = 0.19
P = 0.017
Telomere content (purity corrected for pedBLs)
n = 7
n = 7
n = 39
naïve B cells
GC B cells
pedBL
naïve B cells
GC B cells
leukBL
pleuraBL
solBL
c
P = 0.33
Telomere content tumor/control (log2) – purity corrected
n = 12
n = 27
nonsolid
solid
d
P = 0.093
P = 0.064
P = 0.39
Telomere content tumor/control (log2) – purity corrected
n = 9
n = 3
n = 27
leukBL
pleuraBL
solBL

Supplementary Figure 3. TERT expression and telomere content

a TERT transcript expression in the pedBL cohort (only considering solBL) is significantly higher compared to the expression in normal naive and germinal center (GC) B cell populations.

b Telomere content in pedBL cohort as compared to normal naïve B cells and normal germinal center B cells showing no significantly elevated telomere content in BL as compared to GC B cells. Naive B cells do have a significantly lower telomere content as compared to both, normal GC B cells and pedBL.

c Telomere content in non-solid BL versus solid BL, showing no significant difference between both groups.

d Telomere content across the BL subgroups (leukBL, solBL, and pleuraBL) showing no significant differences between the subgroups.

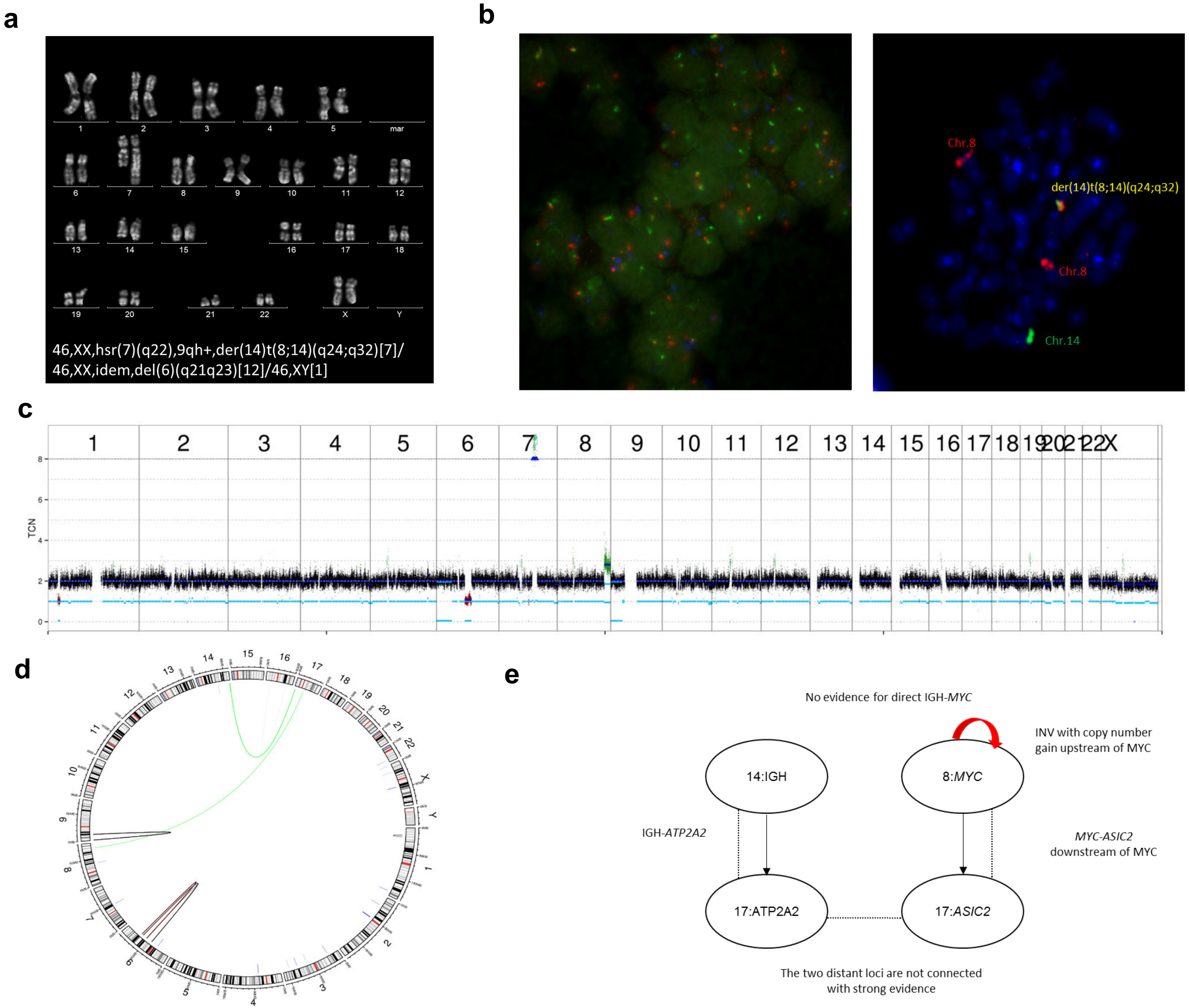
a
46,XX,hsr(7)(q22),9qh+,der(14)t(8;14)(q24;q32)[7]/
46,XX,idem,del(6)(q21q23)[12]/46,XY[1]
b
Chr.8
der(14)t(8;14)(q24;q32)
Chr.8
Chr.14
c
TCN
d
e
No evidence for direct IGH-MYC
INV with copy number gain upstream of MYC
14:IGH
8:MYC
IGH-ATP2A2
MYC-ASIC2 downstream of MYC
17:ATP2A2
17:ASIC2
The two distant loci are not connected with strong evidence

Supplementary Figure 4. Integrative analysis of a complex *MYC-ASIC2*-IGH rearrangement in case 4177434.

a Karyotype showing the der(14)t(8;14)(q24;q32) as consequence of an unbalanced translocation between one IGH locus on chromosome 14 and one *MYC* locus in chromosome 8.

b IGH-*MYC* dual-color, dual fusion probe in interphase nuclei (left) and metaphase (right) cells showing one fusion (yellow) signal in der(14)t(8;14)(q24;q32), two red signals in chromosomes 8 and 1 green signal in chromosome 14.

c Imbalance profile displaying gains (in green) and losses (in red) for each chromosome. Remarkably, the amplicon in chromosomal region 7q includes *CDK6* gene.

d Circle plot indicating structural variants. The green lines represent translocation events, blue lines deletions, red lines duplications, black lines inversions.

e Schematic of the rearrangement events: A three-way translocation involving the IGH-*ASIC2-MYC* genes were detected by WGS. A breakpoint downstream of *MYC* detected by WGS is fused to the *ASIC2* gene locus in chromosome 17. Subsequently, the *ATP2A2* gene also located in chromosome 17 is translocated to the IGH locus.

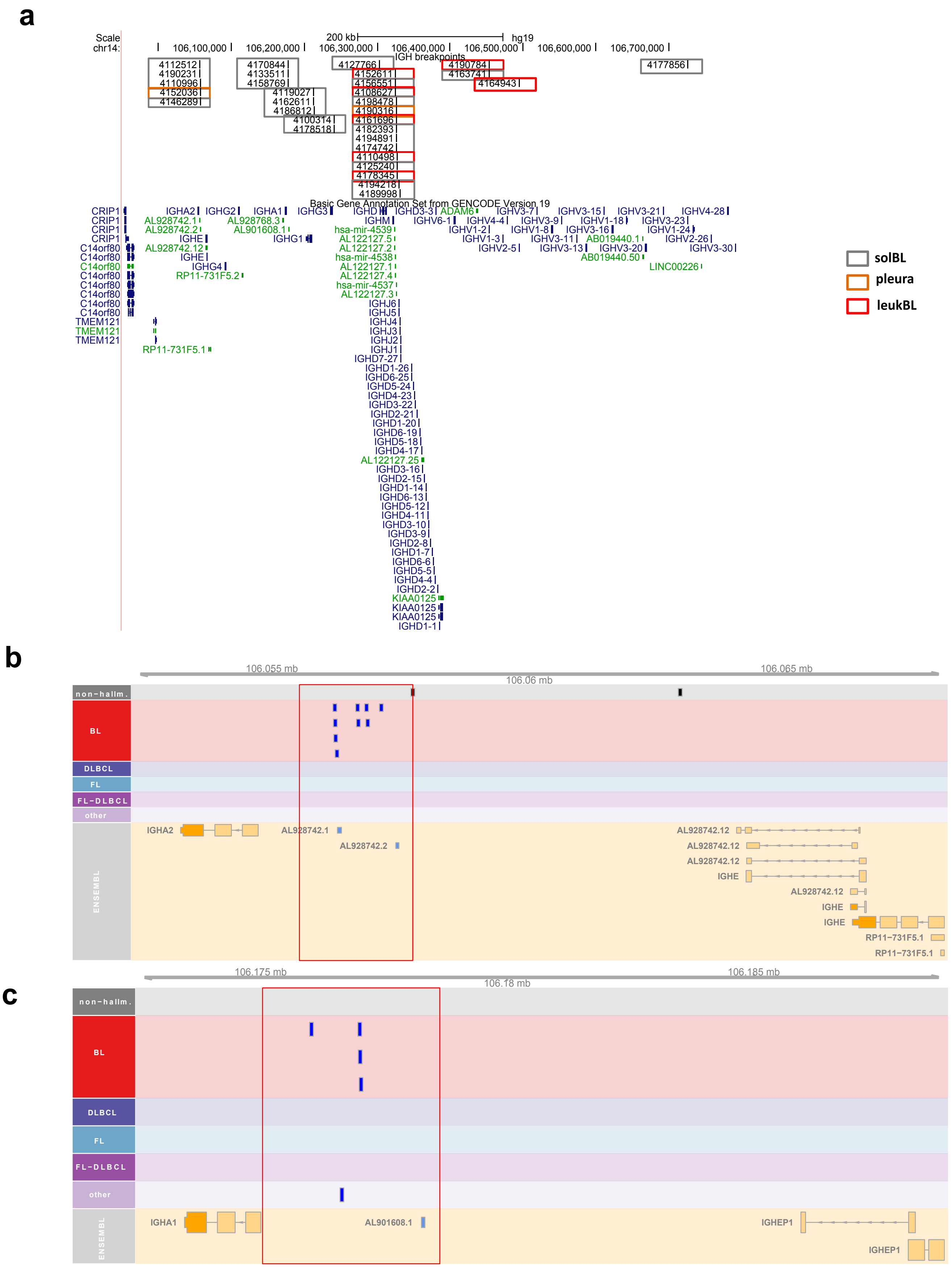

a
Scale
chr14:
200 kb
hg19
106,100,000
106,200,000
106,300,000
106,400,000
106,500,000
106,600,000
106,700,000
IGH breakpoints
4112512
4190231
4110996
4152036
4146289
4170844
4133511
4158769
4119027
4162611
4186812
4100314
4178518
4127766
4152611
4156551
4108627
4198478
4190316
4161696
4182393
4194891
4174742
4110498
4125240
4178345
4194218
4189998
4190784
4163741
4164943
4177856
Basic Gene Annotation Set from GENCODE Version 19
CRIP1
C14orf80
TMEM121
RP11-731F5.1
IGHA2
IGHG2
IGHA1
IGHG3
AL928742.1
AL928742.2
AL928742.12
AL928768.3
AL901608.1
IGHG1
IGHE
IGHG4
RP11-731F5.2
IGHD
IGHM
IGHD3-3
ADAM6
IGHV6-1
IGHV4-4
hsa-mir-4539
hsa-mir-4538
hsa-mir-4537
AL122127.5
AL122127.2
AL122127.1
AL122127.4
AL122127.3
IGHJ6
IGHJ5
IGHJ4
IGHJ3
IGHJ2
IGHJ1
IGHD7-27
IGHD1-26
IGHD6-25
IGHD5-24
IGHD4-23
IGHD3-22
IGHD2-21
IGHD1-20
IGHD6-19
IGHD5-18
IGHD4-17
AL122127.25
IGHD3-16
IGHD2-15
IGHD1-14
IGHD6-13
IGHD5-12
IGHD4-11
IGHD3-10
IGHD3-9
IGHD2-8
IGHD1-7
IGHD6-6
IGHD5-5
IGHD4-4
IGHD2-2
KIAA0125
IGHD1-1
IGHV3-7
IGHV3-9
IGHV1-2
IGHV1-3
IGHV2-5
IGHV1-8
IGHV3-11
IGHV3-13
IGHV3-15
IGHV3-16
IGHV1-18
AB019440.1
AB019440.50
IGHV3-20
IGHV3-21
IGHV3-23
IGHV1-24
IGHV2-26
IGHV4-28
IGHV3-30
LINC00226
solBL
pleura
leukBL
b
106.055 mb
106.06 mb
106.065 mb
non-hallm.
BL
DLBCL
FL
FL-DLBCL
other
ENSEMBL
IGHA2
AL928742.1
AL928742.2
AL928742.12
IGHE
RP11-731F5.1
c
106.175 mb
106.18 mb
106.185 mb
non-hallm.
BL
DLBCL
FL
FL-DLBCL
other
ENSEMBL
IGHA1
AL901608.1
IGHEP1

Supplementary Figure 5. Distribution of breakpoints within the IGH locus.

a Display of the breakpoints within the IGH locus for each case. The IGH-breakpoints of the solBL (grey) and the pleuraBL (orange) were located downstream of the IGHM region, the leukBL (red) had the breakpoints in IGHM or downstream of IGHM region.

b Genomic view showing the IGHA2 region including the switch α2 region (according to ENSEMBL) and displaying the breakpoint positions (some cases harbor more than one breakpoint) in this genomic regions across the ICGC MMML-Seq cohort, including DLBCL, FL, FL-DLBCL, and others like DH-BL lymphoma (Hübschmann et al., under revision). The red square defines the switch region.

c Genomic region showing the IGHA1 region (switch α1) (according to ENSEMBL) and displaying the distribution of the breakpoints (some cases containing more than one breakpoint) in this genomic region across the ICGC MMML-Seq cohort (as describe above). The red square highlights the switch region.

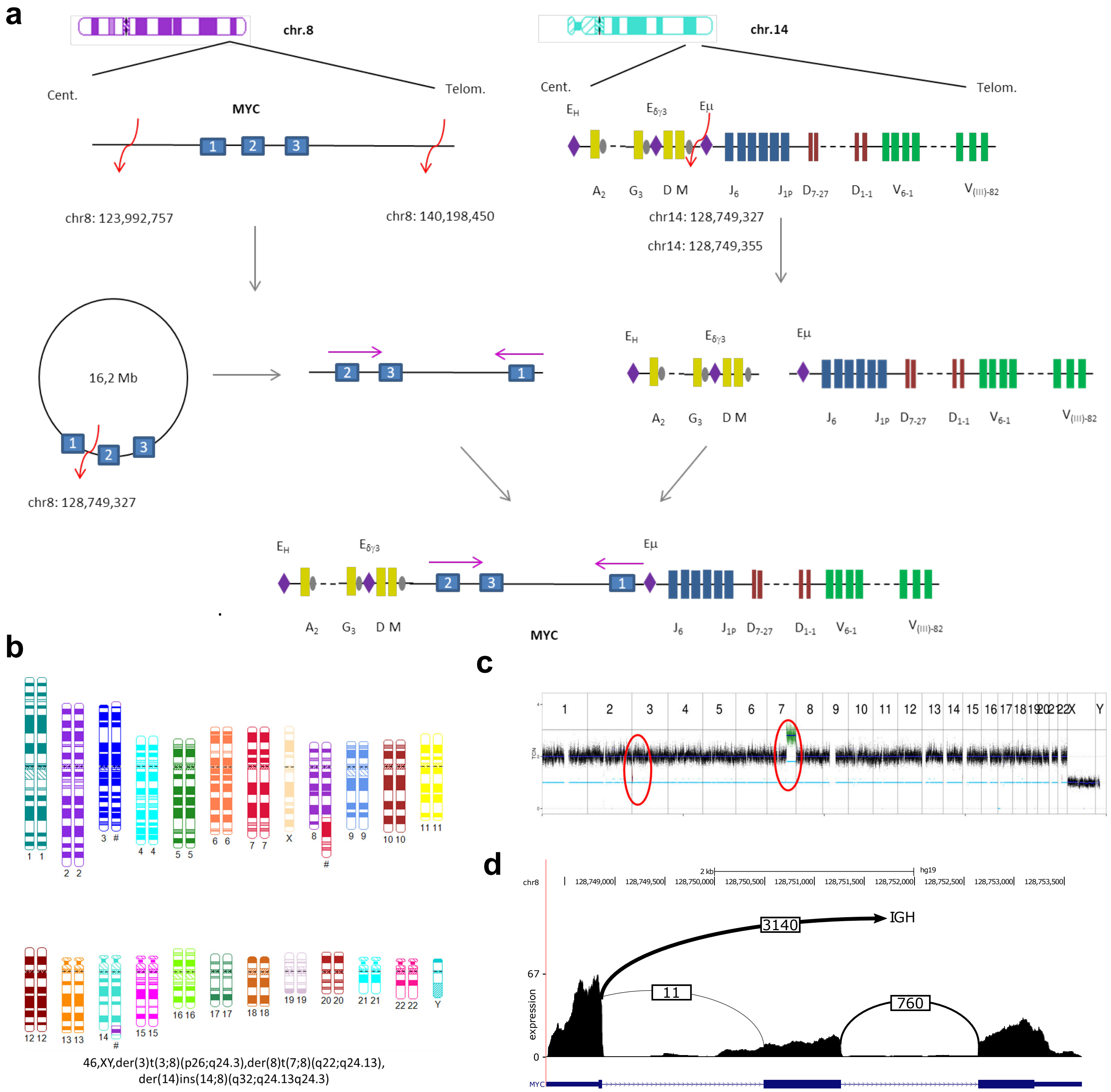

Supplementary Figure 6. Genomic and transcriptomic data of the BL case 4125240

a Representation of an insertion event of *MYC* into the IGH locus. Two breaks, indicated by red arrows upstream and downstream of the *MYC* gene in case 4125240 generated a circular DNA fragment containing the *MYC* gene, which was subsequently opened between exon 1 and 2. The opened fragment contained exon 1 of *MYC* in opposite direction of exons 2 and 3. Insertion of this fragment into chromosome 14 brought *MYC* under the regulation of the enhancers of the IGH locus and lead to the independent expression of exon 2 and exon 3 via Eδγ3, and from exon 1, which is regulated via Eμ enhancer.

b Karyotype predicted using the Cydas online program (http://www.cydas.org/) and based on FISH and structural variant analysis.

c Imbalance profile from WGS data, showing the loss of 3p and gain of 7q (highlighted with the red circles).

d Sashimi plot showing the number of splice reads between exon 1 and exon 2, exon 2 and exon 3, and exon 1 and IGH locus. The low number of splice reads between exon 1 and exon 2 corroborated the data observed by WGS data.

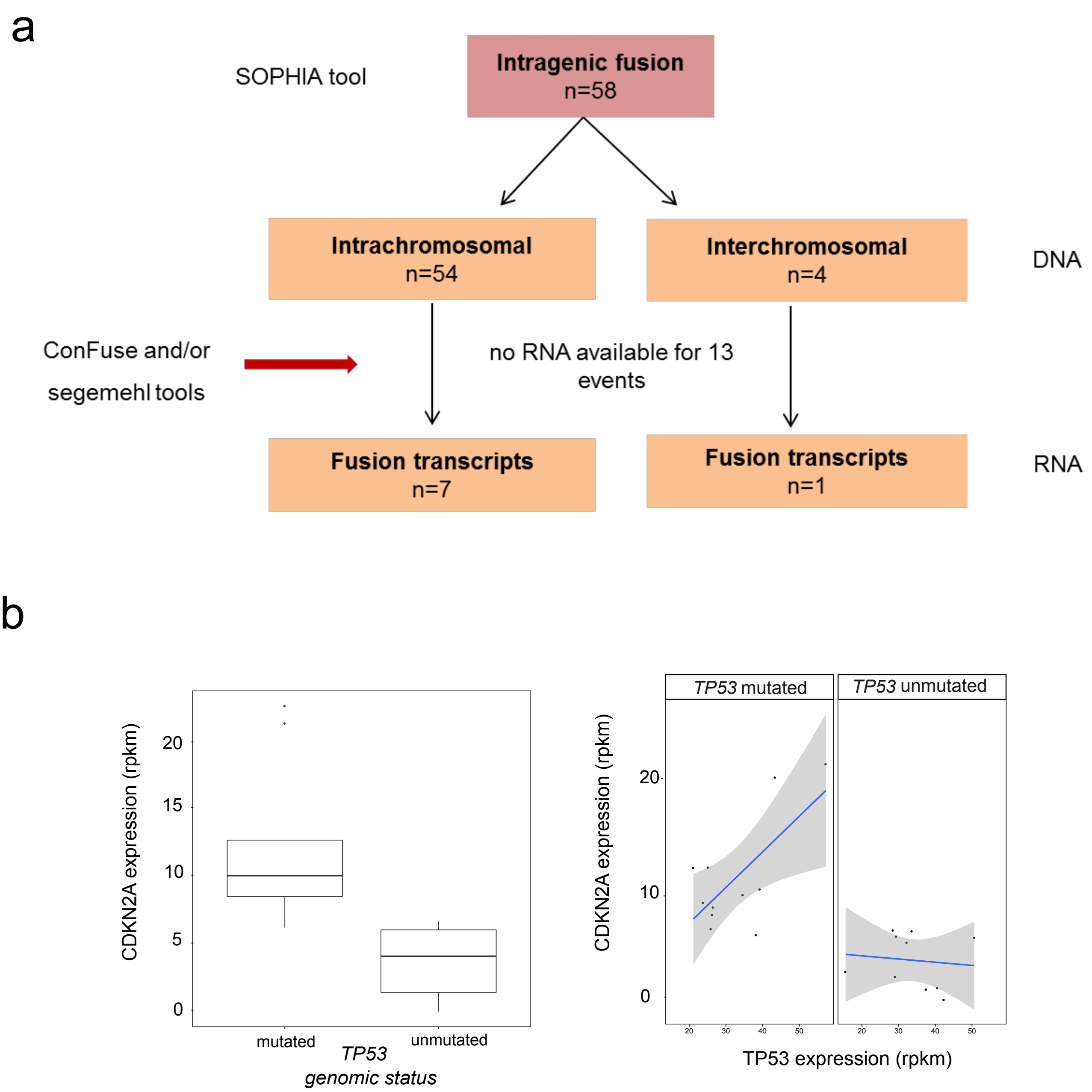

Supplementary Figure 7. RNA-seq analysis

a Schematic work-flow showing the single steps applied for the detection of fusion genes and transcripts. The SOPHIA tool was applied to detect intragenic fusions at the genomic level using WGS data. After that, two independent tools, ConFuse and/or segemehl were applied to search for fusion transcripts at transcriptomic level using RNA-Seq data at the genomic fusions.

b Expression of CDKN2A and TP53 with regard to the *TP53* mutation status in solBL. The box plot on the left displays the CDKN2A expression (RPKM) on the Y axis related to the genomic mutations status of *TP53* on the X axis (mutated n=11, unmutated n= 10). The expression of CDKN2A is higher in solBL cases with *TP53* mutations than without ($p<0.0001$) (Wilcoxon rank sum test). The plot on the right depicts the correlation of TP53 expression (displayed as RPKM on the X axis) and CDKN2A expression (RPKM, Y axis), again separated by TP53 mutation status. Only in cases with *TP53* mutations exhibit a positive correlation ($p=0.021$) (Pearson test).

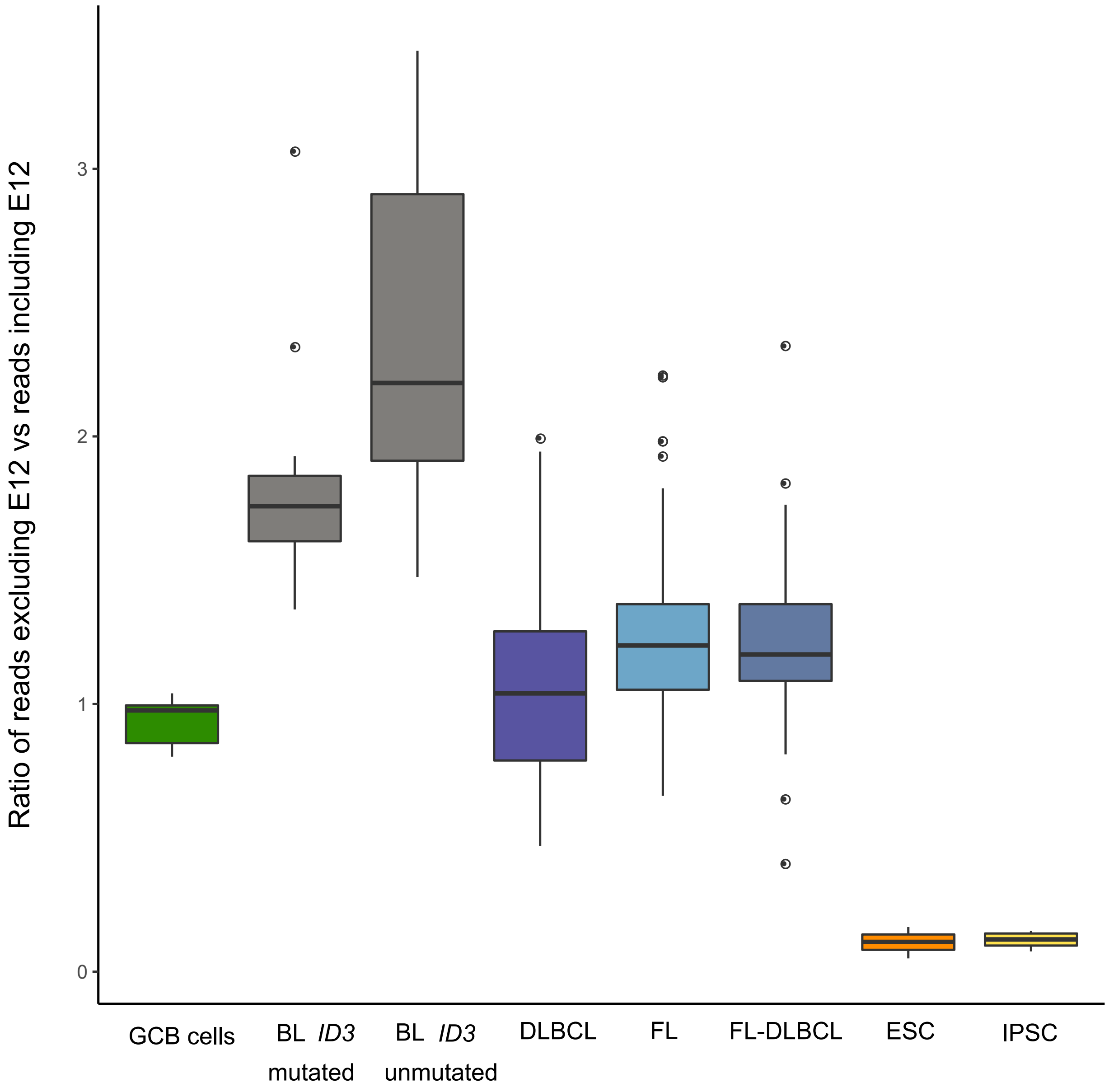

Supplementary Figure 8. TCF3 splicing

Boxplot showing the ratio of spliced reads that are directly spliced to exon E47 vs spliced reads that are spliced to exon E12. While half of the spliced reads splice to E47 in GCB cells (green), most of the BL cases have roughly two thirds of the spliced reads splicing to E47 (grey boxes) (all BL vs GCB cells, $p<0.001$; see Figure 5b for comparison of ID mutated and unmutated BL). A higher fraction of reads excluding E12 than in GCB cells (green) is also observed, though only as a trend, in the non-BL group ($p=0.052$), reaching significance in FL ($n=85$, light blue box, $p=0.008$) and FL-DLBCL ($n=17$, bluish-grey box, $p=0.031$), whereas no significant difference to GCB cells is present in DLBCL ($n=72$, dark blue dark box, $p=0.444$). In contrast, in the ESC ($n=4$, orange) and IPSC ($n=6$, yellow) the splice reads predominately splice to the E12 exon ($p=0.024$ and $p=0.008$, respectively, for comparison to GCB cells). For analyses of DLBCL, FL and FL-DLBCL, in which *ID3* mutations are rare (see Supplementary Figure 9), RNA-seq data of the ICGC MMML-Seq consortium (unpublished), which have been generated using the same sequencing pipeline like the one here applied to BL, were mined. For the analyses of ESC and iPSC we mined data from Friedi et al., 2014[1] and used the alignment tool STAR for mapping splice read. For statistical analysis the Wilcoxon rank sum test was applied and p-values were adjusted for multiple testing according to Benjamini-Hochberg.

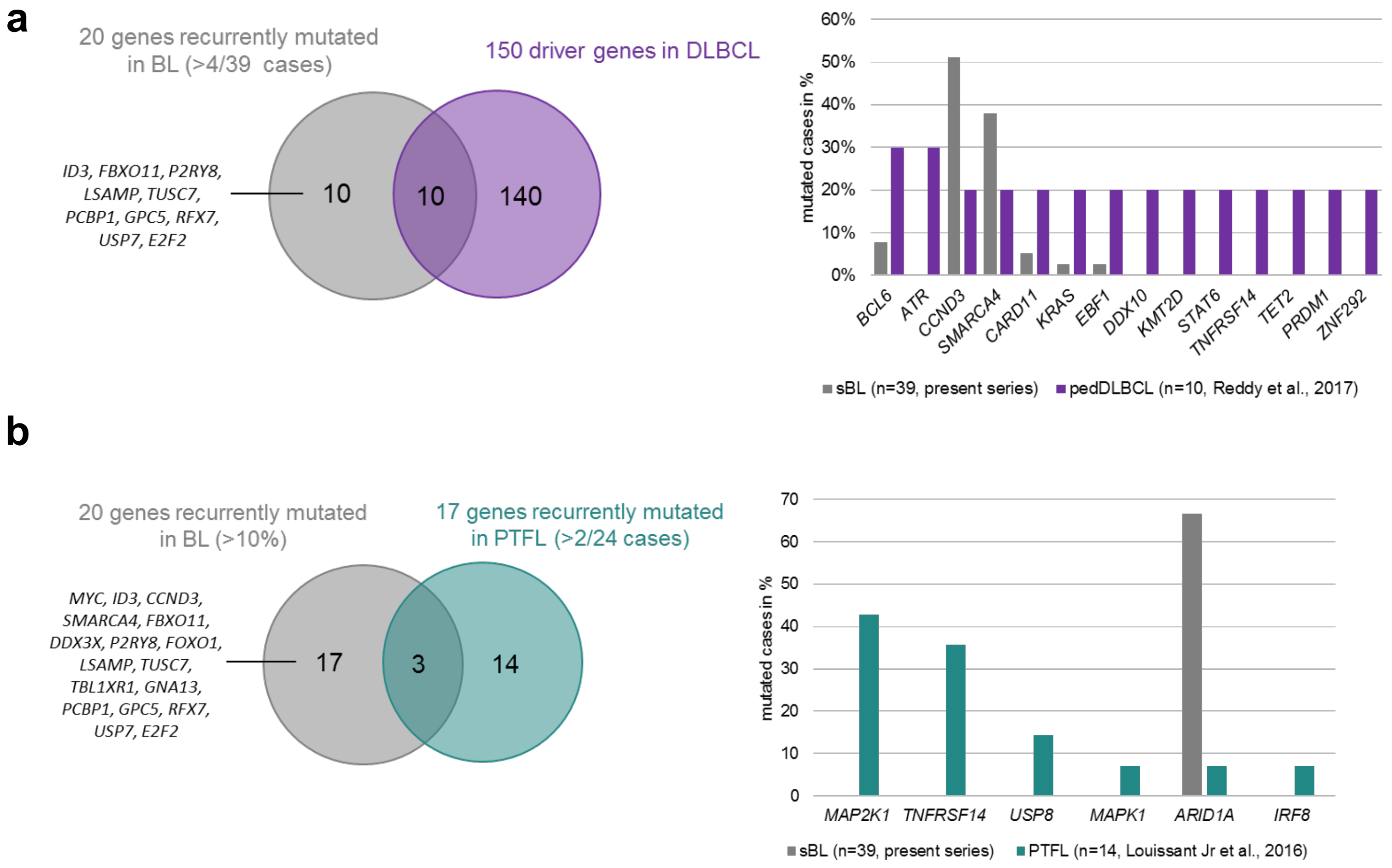

Supplementary Figure 9. Comparison of the mutational landscape of pediatric sBL vs pediatric diffuse large B-cell lymphoma (DLBCL) and pediatric-type follicular lymphoma (PTFL)

a Comparison of recurrently mutated genes in pediatric sBL (n=20) to driver genes (n=150) identified in an overall cohort of 1001 DLBCL by Reddy et al., 2017[2]. This cohort contains a subset of 10 pediatric DLBCL (<u><</u>18 years at diagnosis). On the left side, the Venn diagram shows that only 10 of the 20 recurrently mutated genes in sBL are reported as driver genes in DLBCL. Of those 150 driver genes, only 14 are recurrently mutated in pediatric DLBLC, i.e. affected in <u>></u>2/10 pediatric DLBCL cases. The diagram on the right side shows the relative amount of sBL and pediatric DLBCL cases being mutated in those 14 genes. Eight of these DLBCL driver genes recurrently mutated in pediatric DLBCL are not at all mutated in our sBL cohort and four in less than 10% of the sBL cases.

b Comparison of the mutational landscape of pediatric sBL to mutations in 17 genes identified by Louissaint A Jr et al.[3] as frequently mutated in 24 PTFL (according to Figure 2A in Louissaint A Jr et al., 2016). This cohort contains a subset of 14 PTFL <u><</u>18 years at diagnosis. On the left side, the Venn diagram shows that only 3 of the 20 frequently mutated genes in sBL are recurrently mutated in PTFL. Of the 17 frequently mutated genes in PTFL, only 6 are mutated in PTFL <u><</u> 18 years at diagnosis, i.e. affected in at least 1/14 PTFL <u><</u> 18 years at diagnosis. The diagram on the right side shows the relative amount of sBL and PTFL <u><</u> 18 years at diagnosis being mutated in those 6 genes. Five of these 6 genes are not at all mutated in our sBL cohort.

a

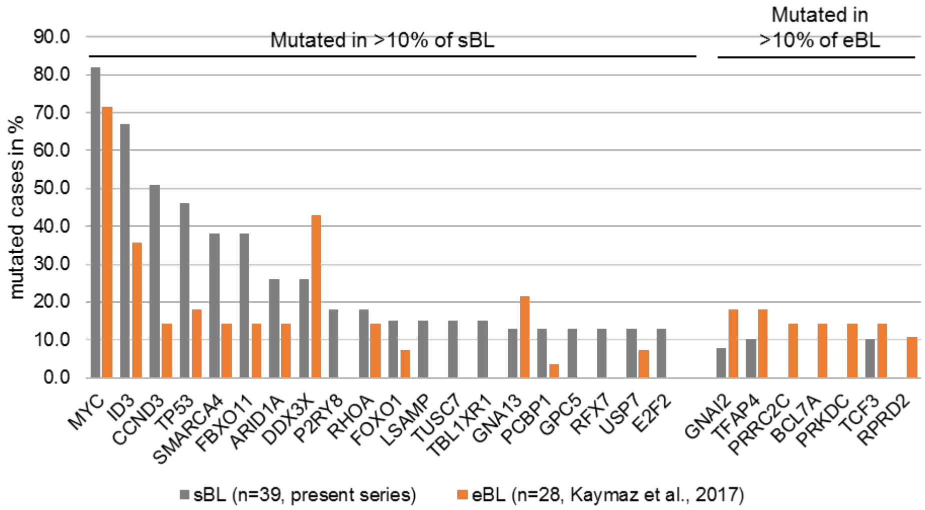

b

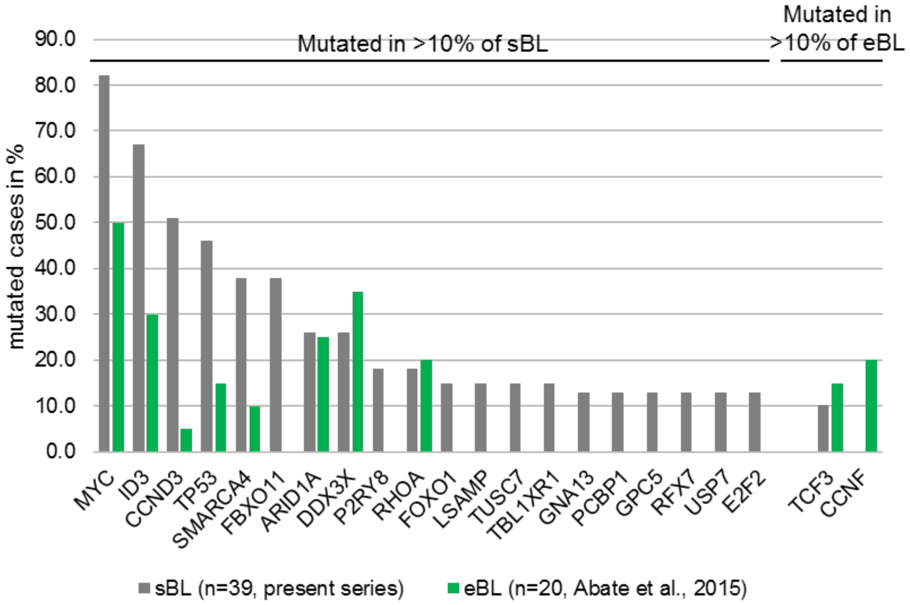

Supplementary Figure 10. Comparison of pediatric sBL vs eBL

a/b Comparison of the frequently mutated genes in sBL (grey) and eBL based on analyses of eBL published by Kaymaz et al. 2017[4] (a, orange) as well as Abate et al., 2015 (b, green). Only genes which were mutated in more than 10% of sBL (left sides) or in more than 10% of eBL (right side) of the respective studies were included. The comparison shows that overall sBL and eBL share a comparable mutational landscape with regard to affected genes, but with some variation in the percentages of cases affected per gene. Whereas eBL harbor a lower amount of cases with mutations in genes like *MYC*, *ID3, CCND3* or *SMARCA4*, they show a higher frequency of mutations in genes like *DDX3X*, *TCF3* or *GNAI2*. These findings are in line with the observations published Kaymaz et al.[4] and Abate et al.[5].

Supplementary Table 1. Clinical and biological features of the cohort.

The table gives an overview of the main clinical characteristics of the cohort and a comparison with previously published cohort from B-NHL a population in BFM trials.

		Overall B-NHL population in NHL-BFM trials*	Whole cohort in this study (n=39)	Subset solBL (n=27)	Subset leukBL (n=9)	Subset pleuraBL (n=3)
**Median age at diagnosis, years [range]**		8.4 [1-18]	8 [2-18]	8 [2-18]	7 [4-10]	11 [7-14]
**Male; n (%)**		873 (87%)	32 (82%)	23 (85%)	7 (78%)	2 (67%)
**St Jude**	**B-AL**	211 (21%)	10 (26%)	1 (4%)	9 (100%)	0 (0%)
	**Stage I**	90 (9%)	1 (2%)	1 (4%)	0 (0%)	0 (0%)
	**Stage II**	221 (22%)	3 (8%)	3 (11%)	0 (0%)	0 (0%)
	**Stage III**	402 (40%)	22 (56%)	19 (70%)	0 (0%)	3 (100%)
	**Stage IV**	80 (8%)	3 (8%)	3 (11%)	0 (0%)	0 (0%)
**CNS involvement (%)**		80 (8%)	4 (10%)	0 (0%)	3 (33%)	0 (0%)
**BM involvement (%)**		261 (26%)	11/39 (28%)	2/27 (7%)	9/9 (100%)	0/3 (0%)
**Events (n)**		129 (13%)	2 (6%)	2 (8%)	0	0

*as published by Burkhardt et al, 2005, British Journal of Haematology, doi:10.1111/j.1365-2141.2005.05735.x
n= number; solBL: solid BL; leukBL: leukemia BL; pleuraBL: pleura BL
CNS = central nervous system
BM = bone marrow
B-AL= Burkitt leukemia
B-NHL= Mature B cell non-Hodgkin lymphoma; NHL-BFM=Non-Hodgkin lymphoma Berlin-Frankfurt-Münster trial

References

1. Friedli, M. *et al*. Loss of transcriptional control over endogenous retroelements during reprogramming to pluripotency. *Genome Res.* **24**, 1251-1259 (2014).

2. Reddy, A. *et al*. Genetic and Functional Drivers of Diffuse Large B Cell Lymphoma. *Cell* **171**, 481-494.e15 (2017).

3. Louissaint, A.,Jr *et al*. Pediatric-type nodal follicular lymphoma: a biologically distinct lymphoma with frequent MAPK pathway mutations. *Blood* **128**, 1093-1100 (2016).

4. Kaymaz, Y. *et al*. Comprehensive Transcriptome and Mutational Profiling of Endemic Burkitt Lymphoma Reveals EBV Type-Specific Differences. *Mol. Cancer. Res.* **15**, 563-576 (2017).

5. Abate, F. *et al*. Distinct Viral and Mutational Spectrum of Endemic Burkitt Lymphoma. *PLoS Pathog.* **11**, e1005158 (2015).
