# Supplementary material for: Genomic and transcriptomic changes complement each other in the pathogenesis of sporadic Burkitt lymphoma
Source: Nat Commun. 2019 Mar 29;10:1459. doi: 10.1038/s41467-019-08578-3 (PMC6440956; doi:10.1038/s41467-019-08578-3)
Supplement: Supplementary file 3 — Description of Additional Supplementary Files [file 41467_2019_8578_MOESM3_ESM.pdf]

Description of Additional Supplementary Files

File Name: Supplementary Data 1
Description: Clinical and molecular characteristics of the analysed BL cohort.

File Name: Supplementary Data 2
Description: Statistics of whole genome sequencing and RNA-Seq.

File Name: Supplementary Data 3
Description: IG-MYC breakpoints and MYC expression pattern of BL cohort.

File Name: Supplementary Data 4
Description: Single nucleotide variants and structural variants detected within the MYC locus.

File Name: Supplementary Data 5
Description: Structural variants in BL cohort (selection of those validated and creating fusion transcripts).

File Name: Supplementary Data 6
Description: Validation of somatic and germline SNVs using PCR and Sanger sequencing.
